# Key body size/shape predictors of physical fitness in Saudi College-aged males: Role of the body roundness index

**DOI:** 10.1371/journal.pone.0333942

**Published:** 2025-10-24

**Authors:** Mohamed Elloumi, Emna Makni, Martin Pacholek, Mehdi Ben Brahim, Ibrahim Aljasser

**Affiliations:** 1 GSD/Health and Physical Education Department, Sport Sciences and Diagnostic Research Group, Prince Sultan University, Riyadh, Saudi Arabia; 2 University of Sousse, Research Laboratory of Exercise Physiology and Pathophysiology (LR19ES09), Faculty of Medicine Ibn Al-Jazzar, Sousse, Tunisia; Università degli Studi di Milano: Universita degli Studi di Milano, ITALY

## Abstract

Physical fitness, a crucial factor in health and well-being, is influenced by an individual’s body composition. This study aimed to identify the key body size/shape predictors of fitness test performances among university-level students with diverse weight categories. This cross-sectional study involved 495 healthy, recreationally active male university students aged 18−23 years, categorized into normal weight (NORMW, n = 256), overweight (OVERW; n = 156), and obese (OB; n = 124) groups based on their body mass index (BMI). Anthropometric measurements including weight, height, BMI, waist and hip circumference (WC and HC), waist-to-hip and waist-to-height ratios (WHR and WHtR), body roundness and shape body indexes (BRI and ABSI) were recorded. The shuttle-run, push-ups, 20m Multi-Stage shuttle-run, and sit-and-reach tests were performed. The OB and OVERW groups performed significantly lower fitness test performances than the NORMW group (medium-to-large effect size). The correlation matrix showed that all anthropometric measures correlated with test performances, except for the sit-and-reach in the OB group and the shuttle-run in the NORW group. Higher WHtR and HC improved shuttle-run performance predictability in the OB group (R^2^ = −0.29), while WC and HC, and WC and BMI improved push-up predictability in the OB (R^2^ = −0.38) and OVERW (R^2^ = −0.24) groups. BRI was the best indicator of VO_2_max performance, accounting for 56%, 42%, and 32% of its variance in OB, OVERW, and NORMW groups, respectively. The BRI is proposed as a potential alternative to BMI for evaluating cardiorespiratory endurance performance, enabling individual monitoring.

## Introduction

Physical fitness is a key determinant of health and well-being through several health-related fitness components. It includes cardiorespiratory endurance, muscle strength, muscle endurance, flexibility, and body composition [1]. It is well acknowledged that a person’s body size and shape substantially impact their physical ability and athletic potential [2–4]. Understanding the relationship between body size, shape, and health-related fitness components is crucial for health risk assessments, training program optimization, individual exercise design, and public health strategies. In this context, numerous investigations have been undertaken to ascertain the major determinants of body size and body shape indices of physical fitness in various populations [4–8]. Body weight classification, including overweight and obesity, is typically determined by the body mass index (BMI); calculated by dividing weight in kilograms by height in meters squared. BMI has become the commonly used anthropometric predictor for health-related physical fitness [2,4,5,7,8]. Indeed, Ben Brahim et al [5] indicated that BMI was the best indicator of performance in jump, sprint, agility, and cardiorespiratory endurance tests in healthy students. Moreover, Prieto-González [2] reported weak to moderate significant correlations between BMI and standing long jump and shuttle-run tests in college-aged males. Shalabi et al [4] found that BMI significantly influences both muscular strength and flexibility of female Saudi students. However, BMI’s reliability and accuracy in predicting physical fitness are frequently questioned due to its insufficient ability to distinguish between lean and fat mass, and to assess fitness and metabolic health [9,10]. Waist and hip circumferences (WC and HC), waist-to-hip ratio (WHR), and waist-to-height ratio (WHtR) are considered indicators of central obesity and health-related fitness components due to their relationship with fat distribution and BMI limitations [11,12]. Prieto-González [2] and Ben Brahim et al [3] stated weak significant correlations between WHR and jumping performance in college-aged males. Similarly, Shalabi al [4] reported that WC significantly correlated with the physical activity score but not with health-related physical fitness in female Saudi students. Also, Reyes-Ferrada et al [13] found a negative correlation between cardiorespiratory fitness and plasma atherogenic index, with elevated WHtR influencing about one-third of this relationship.

More recently, alternative anthropometric indices like the body shape index (ABSI) and body roundness index (BRI) have been introduced to better reflect health status [14–17]. BRI, developed by Thomas et al [17] accurately estimates visceral and total body fat percentages using elliptical models based on human body shape, eccentricity, waist circumference, height, and weight. It also considers waist circumference in addition to weight and height. The relationship between ABSI and BRI and health outcomes is growing [14–18], but the connection between these body shape indexes and physical fitness in healthy recreational individuals remains unexplored.

Given the inconsistent results regarding the predictive power of a single body size and body shape of physical performance in adults with different body categories, a multi-faceted approach is recommended [19]. Identifying these factors is crucial for promoting balanced and sustainable health, especially when dealing with diverse body composition populations. Furthermore, to our knowledge, the possible relationship between BRI and ABSI and health-related physical fitness in recreationally active adults from different body categories has not been verified, and no prediction model has been obtained. Accordingly, this study aims to identify key body size and shape indexes that predict field fitness test performance in collegiate male adults based on their body mass category.

## Method

### Procedure

**Fig 1:**
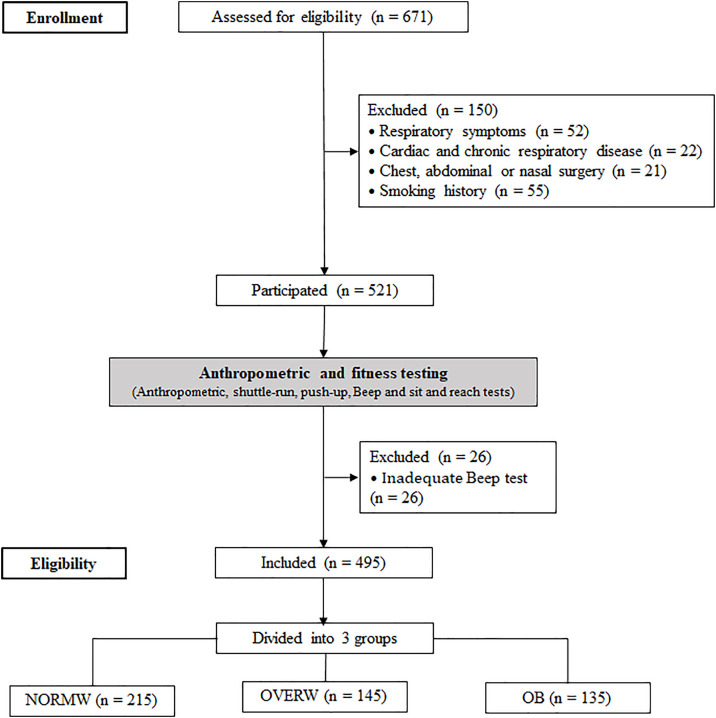
Chart flow of the study.

A cross-sectional study was conducted on collegiate Saudi males to explore the causal relationships and potential mechanisms between physical fitness performance and key body size and shape determinants. This model investigated whether other body shapes/sizes beyond BMI determine students’ physical fitness. The outcome measures included anthropometrics, push-ups, shuttle-run, flexibility, and 20m Multi-Stage Shuttle-run tests. Participants conducted anthropometric measurements, shuttle-run, push-ups, sit-and-reach, and endurance tests at sports facilities three times on non-consecutive days following technical specifications and the “National Student Physical Health Standard” (Fig 1). Participants were instructed to refrain from physical activity for at least 48 hours before the testing. During the testing sessions, they underwent a standardized 15-minute warm-up consisting of progressive running, dynamic stretching and joint mobility, jump, and progressive sprints. Standard verbal encouragement ensured maximal effort throughout was provided for all participants.

### Participants

Four hundred and ninety-five recreationally active male university students aged 18–23 (19.1 ± 1.1 years; weight: 81.9 ± 22.1 kg; height: 1.73 ± 0.1 m) voluntarily participated in the study. The sample consisted of rural and urban students, selected using a standard questionnaire based on specific exclusion criteria [20]:

(1) Respiratory symptoms within the past four months before testing (n = 52); (2) Cardiac disease, acute or chronic respiratory diseases like chronic cough, congestion, phlegm, wheezing, or asthma (n = 22); (3) Chest, abdominal, or nasal surgery (n = 21); and (4) smoking (n = 55). Based on these criteria, 150 students out of 671 were excluded. The tests were conducted on 521 students, with 26 excluded due to inadequate performance in the 20m Multi-Stage Shuttle-run test. The study involved 495 students, categorized into three groups based on their body weight, using World Health Organization criteria [21]: A normal-weight individual is defined as having a BMI between 18.5 and 24.9 kg.m^-2^ (n = 215). In contrast, an overweight individual has a BMI ≥ 25 kg.m^-2^ (n = 145). An individual with obesity has a BMI ≥ 30 kg.m^-2^ (n = 135). The sample size was calculated using the following formula [22]:


$$n=N^{*}X/\;(X+N\;–\;\text{1}),\mathrm{\backslash ]}$$

Where, X = Z_α/2_^2^-*p*(1-p)/MOE^2^, Z_α/2_ is the critical value of the Normal distribution at α/2, MOE is the margin of error, p is the sample proportion, and N is the population size.

The confidence level was 99% with a 5% confidence interval, and the probability of success was chosen at 50% to maximize the sample size. A minimum of 284 participants is needed to obtain a representative population sample.

Participants provided written consent before the study after being informed about its purpose, testing procedures, and potential risks. The study, approved by the Institutional Review Board of the Bioethics Committee at Prince Sultan University (PSU IRB-2024-10-0188), complied with the Helsinki Declaration 2013 and commenced on November 3, 2024, and concluded on January 12, 2025.

### Anthropometrics

Weight and height were measured using an electronic scale and a standing stadiometer (Seca Instrument Ltd., Hamburg, Germany), with a precision of 0.1 kg and 0.1 cm, respectively. BMI (kg.m^-2^) was calculated by dividing body weight (kg) by the square of height (m). The waist and hip circumferences (WC and HC) were measured using a ribbon meter, and the waist/hip and waist/height ratios (WHR and WHtR) were calculated. The body shape index (ABSI) and body roundness index (BRI) were also calculated using the corresponding formulas [14,17]: ABSI = WC/ BMI^2/3^ × height^1/3^ and BRI = 364.2 − 365.5 × √(1 − [WC/ 2π]^2^/ [0.5 × height]^2^).

The height and WC are in centimeters, while BMI is calculated by dividing weight in kilograms by height in squared meters.

### Physical fitness tests

The physical fitness tests included shuttle-run, push-ups, sit-and-reach, and a 20m multi-stage fitness test. These tests are routinely conducted as part of the students’ practical activities.

### Shuttle-run test

Agility was assessed through a 4 × 10 m shuttle-run with two parallel lines drawn 10 m apart on the ground using photocells (HL3-1x Wireless Photocell-TAG-Heuer Professional Timing). Participants were asked to sprint 40 meters, crossing each line with both feet, while picking up and carrying alternately the two sponges behind the lines back to the starting line [23]. The photocells were placed at waist height and the time was recorded as a hundredth of a second. Standing 0.5 meters behind the starting line, players started sprints promptly and were encouraged to perform each sprint as quickly as possible. Participants initiated the test from a standing position 0.5 meters behind the starting line, aiming to run as fast as possible. After one practice trial, they had two attempts separated by a 3-minute rest. The best performance was selected for analysis.

### Push-up

The upper body’s muscular strength was evaluated through a push-up test, which was measured by the number of push-ups performed per minute. Participants should face the ground, with their hands slightly wider than their shoulders, and then extend their legs backward to balance on their hands and toes. From this position, participants inhale by bending their elbows 90 degrees, then exhale by contracting chest muscles and raising their hands, returning to the starting position [24]. The test was conducted twice and the best performance was retained for analysis.

### Sit-and-reach

This test was designed to assess flexibility, specifically hamstring extensibility [25], using a Baseline Sit-and-reach Trunk Flexibility Assessment Testing Box (Sacramento, CA, USA). As previously described [2], the test involved a participant seated on a floor with knees extended, feet flat against a sit-and-reach box and hands on top. They reached forward slowly, bending their trunk and keeping their knees straight. The researcher measured the distance their fingers reached from their toes, and the results were rounded to the nearest 0.5 cm. Positive results were obtained when participants reached beyond their toes, while negative results were obtained when they couldn’t [26]. The test was conducted twice and the best performance was retained for analysis.

### 20-m Multi-Stage shuttle-run test

This test was used to estimate cardiorespiratory endurance and maximal oxygen uptake (VO_2_max). As previously described [2], the test is a back-and-forth incremental, continuous, maximal-to-fatigue running test on a 27 m wide surface. Participants ran between two lines set 20 m apart at a pace dictated by audio. The initial speed was 8.5 km h^-1^, and it increased by 0.5 km h^-1^ every minute. Participants were required to step behind the 20 m line when tones were emitted. If the line was not reached before the sound, they were given one warning and had to continue running to catch up within two more tones. The test ended when participants received the second warning or stopped due to fatigue. The athlete’s score was the number of shuttles completed before finishing the test [27]. VO_2_max was calculated using the following formula [27]: VO_2_max = 0.0276x + 27.504.

### Statistical analyses

The results are presented as average ± SD. After checking the normality of the variables using the Kolmogorov-Smirnov test, the one-way ANOVA was carried out to compare the data of the three groups. The partial eta-squared (ηp^2^) for effect size was calculated for each variable (ηp^2^ up to 0.059 = small; between 0.059 and 0.138 = medium, and greater than 0.138 = large; [28]).

Pearson’s regression analysis was conducted to investigate the association between the assessed variables. Cohen’s classification of correlation coefficient is characterized into weak (0.10 ≤ r < 0.30), moderate (0.30 ≤ r < 0.50), and strong (r ≥ 0.50) [29].

First, we used stepwise and maximum R^2^ improvements to identify variables that could predict the different test performances for each group separately. The multiple regression models included anthropometry, body size, and shape measurements as independent variables for each group’s physical fitness test performance. The coefficients of determination (R^2^), standard error (SE), and residual standard deviation (RSD) were calculated for each group. Statistical analyses were conducted using the SPSS package (SPSS Inc. Chicago. IL. Version 26.0), with a significance threshold of p < 0.05.

## Results

### Anthropometric and fitness test results

Table 1 presents the anthropometric data and physical fitness test performances for the three groups. Obese (OB) participants have higher weight, BMI, WC, HC, WHR, WHtR, and BRI, and lower physical test performance compared to overweight (OVERW) and normal weight (NORMW) individuals. Similarly, OVERW participants have higher weight, BMI, WC, HC, WHR, WHtR, and BRI, and lower physical test performance than NORMW individuals. The values of ηp^2^ revealed significant differences in weight, BMI, WC, HC, WHtR, BRI, push-up and VO_2_max tests among the different groups, while small effect sizes were observed for age, height, WHR, ABSI, shuttle-run and sit-and-reach tests.

**Table 1:** Anthropometric and test performance data of the participants (mean ± SD).

	OB (n = 135)	OVERW (n = 145)	NORMW (n = 215)	ηp^2^/p value
Age (year)	19.01 ± 1.09	19.2 ± 1.2	19.03 ± 1.01	0.005/0.33
Weight (kg)	110.2 ± 17.7**††	82.2 ± 7.0**	63.80 ± 7.79	0.74/ < 0.001
Height (m)	1.74 ± 0.08	1.74 ± 0.06	1.73 ± 0.06	0.007/0.19
BMI (kg.m^-2^)	36.4 ± 5.6**††	27.2 ± 1.3**	21.32 ± 2.12	0.77/ < 0.001
WC (cm)	114.3 ± 11.9**††	93.5 ± 8.3**	79.4 ± 8.4	0.70/ < 0.001
HC (cm)	120.0 ± 11.6**††	105.2 ± 6.4**	93.0 ± 7.5	0.63/ < 0.001
WHR	0.96 ± 0.09**†	0.93 ± 0.12**	0.87 ± 0.12	0.10/ < 0.001
WHtR	0.66 ± 0.07**††	0.55 ± 0.05**	0.46 ± 0.05	0.68/ < 0.001
BRI	5.77 ± 0.89**††	4.62 ± 0.60**	3.70 ± 0.54	0.62/ < 0.001
ABSI	0.079 ± 0.007	0.080 ± 0.007	0.079 ± 0.008	0.003/0.50
Push-up test (N)	15.6 ± 8.9**††	22.5 ± 9.9**	30.1 ± 9.1	0.30/ < 0.001
Shuttle-run test (s)	13.23 ± 1.18**††	12.36 ± 3.26**	11.56 ± 0.74	0.11/ < 0.001
VO_2_max (ml.kg^-1^.min^-1^)	32.2 ± 4.7**††	36.8 ± 4.42**	39.1 ± 5.1	0.25/ < 0.001
Sit-and-reach test (cm)	−0.72 ± 8.7**	0.9 ± 9.4*	2.9 ± 10.1	0.03/ < 0.05

Abbreviations: OB, obese; OVERW, overweight; NORMW, normal weight; ABSI, a body shape index; BMI, body mass index; BRI, body roundness index; HC, hip circumference; WC, waist circumference; WHR, waist-to-hip ratio; WHtR, waist-to-height ratio; VO_2_max, maximal oxygen intake.

*: Significant difference with NORMW group, *p < 0.05, **p < 0.01; †: Significant difference with OVERW group, †p < 0.05, ††p < 0.01.

Table 2 summarizes the correlation matrix between body size/shape and physical fitness test performances for the three groups. The shuttle-run performance significantly correlated with all anthropometric measures in the OB group, except height, WHR, and ABSI (r = 0.42 to r = 0.50; p < 0.01), and only with weight and BMI (r = 0.16 and r = 0.20; p < 0.05 and p < 0.01, respectively) in the OVERW group. The NORMW group showed no significant correlation between shuttle-run performance and anthropometric measurements.

**Table 2:** Correlation matrix between body size and shape and physical test performance in the three population groups.

OB (n = 135)				
	Shuttle-run test (s)	Push-up test (N)	VO_2_max (ml/min/kg)	Sit-and-reach test (cm)
Weight (kg)	0.42**	−0.53**	−0.31**	−0.14
Height (m)	−0.07	−0.11	0.34**	−0.13
BMI (kg.m^-2^)	0.50**	−0.49**	−0.53**	−0.08
WC (cm)	0.48**	−0.60**	−0.60**	−0.19*
HC (cm)	0.45**	−0.49**	−0.35**	−0.15
WHR	0.05	−0.15	−0.31**	−0.06
WHtR	0.50**	−0.55**	−0.73**	−0.14
BRI	0.46**	−0.43**	−0.75**	−0.07
ABSI	0.12	−0.20*	−0.17*	−0.10
OVERW (n = 145)				
	Shuttle-run test (s)	Push-up test (N)	VO_2_max (ml/min/kg)	Sit-and-reach test (cm)
Weight (kg)	−0.04	−0.33**	−0.01	−0.17*
Height (m)	0.01	−0.20*	0.26**	−0.09
BMI (kg.m^-2^)	−0.08	−0.33**	−0.36**	−0.17*
WC (cm)	0.01	−0.46**	−0.41**	−0.16*
HC (cm)	−0.07	−0.24**	−0.09	−0.02
WHR	−0.03	−0.24**	−0.44**	−0.12
WHtR	−0.03	−0.40**	−0.62**	−0.13
BRI	−0.03	−0.30**	−0.65**	−0.08
ABSI	−0.04	−0.34**	−0.48**	−0.10
NORMW (n = 215)				
	Shuttle-run test (s)	Push-up test (N)	VO_2_max (ml/min/kg)	Sit-and-reach test (cm)
Weight (kg)	0.16*	−0.32**	−0.15*	−0.13
Height (m)	0.01	−0.20*	0.19*	−0.27**
BMI (kg.m^-2^)	0.20**	−0.26**	−0.32**	0.03
WC (cm)	0.05	−0.26**	−0.44**	−0.21*
HC (cm)	0.07	−0.14	−0.23**	0.02
WHR	0.02	−0.12	−0.22**	−0.20*
WHtR	0.06	−0.20*	−0.52**	−0.13
BRI	0.05	−0.13	−0.56**	−0.04
ABSI	−0.061	−0.07	−0.31**	−0.21*

Abbreviations: OB, obese; OVERW, overweight; NORMW, normal weight; ABSI, a body shape index; BMI, body mass index; BRI, body roundness index; HC, hip circumference; WC, waist circumference; WHR, waist-to-hip ratio; WHtR, waist-to-height ratio; VO_2_max, maximal oxygen intake.

*p < 0.05, **p < 0.01

The push-up performance was significantly correlated with all anthropometric measures in the OB group (r = 0.20 to r = 0.60; p < 0.05 to p < 0.01), except height and WHR, and with all anthropometric measures in the OVERW group (r = 0.20 to r = 0.46; p < 0.05 to p < 0.01). The NORMW group’s push-up performance was significantly correlated with weight, height, BMI, WC, and WHtR (r = 0.20 to r = 0.32; p < 0.05 to p < 0.01).

The VO_2_max was significantly correlated with all anthropometric measures in the OB group (r = 0.17 to r = 0.75; p < 0.05 to p < 0.01), except age, and with all anthropometric measures in the OVERW group (r = 0.24 to r = 0.65; p < 0.05 to p < 0.01), except weight and HC. The NORMW group’s VO_2_max was significantly correlated with all anthropometric measures (r = 0.15 to r = 0.56; p < 0.05 to p < 0.01).

The sit-and-reach test performance was weakly correlated with WC in the OB group (r = 0.19; p < 0.05), and with weight and BMI in the OVERW group (r = 0.17; p < 0.05). The NORMW group’s sit-and-reach performance was also weakly correlated with height, WC, WHtR, and ABSI (r = 0.27, r = 0.21, r = 0.20, and r = 0.21, respectively; p < 0.05 to p < 0.01).

The multiple regression models between physical test performances and independent variables are presented in Tables 3–5.

**Table 3:** Multiple regression models predicting shuttle-run performance in the three population groups.

Obese (n = 135)					
Independent variables	NSCC	R^2^	RSD	SE	P
Constant	6.29	0.29	1.01	0.97	<0.01
WHtR	0.37**				
HC (cm)	0.24**				
The equation for shuttle-run performance: 0.37 × WHtR + 0.24 × HC + 6.29.					
Overweight (n = 145)					
No significant multiple regression model					
Normal weight (n = 215)					
Independent variables	NSCC	R^2^	RSD	SE	P
Constant	10.06	0.11	8.88	0.51	<0.05
BMI (kg.m^-2^)	−0.20*				
The equation for shuttle-run performance: −0.20 × BMI + 10.06.					

Abbreviations: OB, obesity; OVERW, overweight; NORMW, normal weight; BMI, body mass index; HC, hip circumference; WHtR, waist-to-height ratio; NSCC, non-standardized correlation coefficient; RSD, residual standard deviation; SE, standard error.

*p < 0.05, **p < 0.01.

**Table 4:** Multiple regression models predicting push-up performance in the three population groups.

Obese (n = 135)					
Independent variables	NSCC	R^2^	RSD	SE	P
Constant	74.28	0.38	7.13	6.87	<0.01
WC (cm)	−0.48**				
HC (cm)	−0.18*				
Equation for push-up performance: −0.48 × WC −0.18 × HC + 74.28.					
Overweight (n = 145)					
Independent variables	NSCC	R^2^	RSD	SE	P
Constant	102.20	0.24	8.72	15.17	<0.01
WC (cm)	−0.39**				
BMI (kg.m^-2^)	−0.18*				
Equation for push-up performance: −0.39 × WC −0.18 × BMI + 102.20.					
Normal weight (n = 215)					
Independent variables	NSCC	R^2^	RSD	SE	P
Constant	53.74	0.10	8.63	4.87	<0.05
Weight (kg)	−0.32**				
Equation for push-up performance: −0.32 × Weight + 53.74.					

Abbreviations: OB, obesity; OVERW, overweight; NORMW, normal weight; BMI, body mass index; HC, hip circumference; WC, waist circumference; NSCC, non-standardized correlation coefficient; RSD, residual standard deviation; SE, standard error.

*p < 0.05, **p < 0.01.

**Table 5:** Multiple regression models predicting VO_2_max performance in the three population groups.

Obese (n = 135)					
Independent variables	NSCC	R^2^	RSD	SE	P
Constant	54.79	0.56	3.11	1.77	<0.01
BRI	−0.75**				
The equation for VO_2_max performance: −0.75 × BRI + 54.79.					
Overweight (n = 145)					
Independent variables	NSCC	R^2^	RSD	SE	P
Constant	75.09	0.46	3.28	5.65	<0.01
BRI	−0.59**				
BMI (kg.m^-2^)	−0.20*				
The equation for VO_2_max performance: −0.59 × BRI – 0.20 × BMI + 75.09.					
Normal weight (n = 215)					
Independent variables	NSCC	R^2^	RSD	SE	P
Constant	58.82	0.31	4.34	2.04	<0.01
BRI	−0.56**				
The equation for VO_2_max performance: −0.56 × BRI + 58.82.					

Abbreviations: OB, obesity; OVERW, overweight; NORMW, normal weight; BMI, body mass index; BRI, body roundness index; HC, hip circumference; WHR, waist-to-hip ratio; NSCC, non-standardized correlation coefficient; RSD, residual standard deviation; SE, standard error.

*p < 0.05, **p < 0.01.

The results of the multiple regressions showed that WHtR and HC were significant predictors of shuttle-run performance in the OB group (R2 = 0.29 (r = 0.54); p < 0.01), while BMI was a significant predictor in the NORMW group (R2 = 0.11 (r = 0.33); p < 0.05). No significant multiple regression model was found in the OVERW group.

The results of the multiple regressions showed that WC and HC were significant predictors of push-up test performance in the OB group (R^2^ = 0.38 (r = 0.62); p < 0.01), while WC and BMI were significant predictors in the OVERW group (R^2^ = 0.24 (r = 0.49); p < 0.01). Weight was the only significant predictor in the NORMW group (R^2^ = 0.10 (r = 0.32); p < 0.05).

The results of the multiple regressions showed that BRI was a significant predictor of VO_2_max in the OB group (R^2^ = 0.56 (r = 0.75); p < 0.01), while BRI and BMI were significant predictors in the OVERW group (R^2^ = 0.49 (r = 0.68); p < 0.01). BRI was the only significant predictor in the NORMW group (R^2^ = 0.31 (r = 0.56); p < 0.01).

Multiple regressions revealed that WC is a weak predictor of sit-and-reach test performance in the OB group, while BMI, height, and ABSI are weak predictors in the OVERW group.

## Discussion

This study aimed to identify the body size/shape factors predicting performance in various fitness tests, including the shuttle-run, push-ups, sit-and-reach test, and 20-m Multi-stage shuttle-run test among university-level students with diverse body composition. The results demonstrated that the OB group performed poorly in agility (shuttle-run), muscular endurance (push-ups), cardiorespiratory endurance (20-m Multi-stage shuttle-run), and flexibility (sit-and-reach) tests, and had higher measurements for weight, BMI, WC, HC, WHR, WHtR, and BRI compared to their OVERW and NORMW counterparts. Similar trends were observed in the OVERW group compared to the NORMW group. The findings of this study support previous research in young adults, indicating that excessive body weight negatively impacts fitness performance, highlighting the expected association between fitness and excessive body weight [3,6,19,30].

The correlation matrix revealed that body size, shape, and physical test performance negatively affect fitness outcomes in the OB group, except for flexibility, with WC being the only measure significantly correlated with sit-and-reach test performance. Chen et al [6] found that flexibility is negatively associated with WC and abdominal obesity risk in both men and women, despite previous research suggesting no correlation between BMI and WC and flexibility performance among obese students [2]. Obesity can cause mechanical restrictions, inflammation, sedentary lifestyles, postural changes, and reduced muscle elasticity, impacting trunk, hips, and lower body movement [31], with age-influencing flexibility assessment.

The OVERW group showed significant correlations between push-up and cardiorespiratory endurance performances, with height positively affecting cardiorespiratory endurance but slightly impacting push-up performance. The study also revealed that weight, BMI, height, WC, and WHtR affect shuttle-run and push-up test performances.

The NORMW group, with flexibility weakly influenced by weight and WC. Cardiorespiratory endurance is correlated with body size and shape, with higher correlations for WC, WHtR, and BRI. While the impact of excess weight and central fat is less pronounced in the normal-weight group compared to the overweight and obese groups, these factors still negatively affect muscular endurance, cardiorespiratory endurance, and flexibility. Agility, however, remains minimally affected in this group. Overall, muscular endurance correlated negatively with higher body mass and central fat, while agility and flexibility showed weak or no significant correlation with anthropometric measures. Excessive body mass negatively impacts agility performance. Studies by Fogelholm et al [32] and Lockie et al [30] showed that participants with higher body mass and WHR achieve poorer performance outcomes in push-ups than those with lower WHR values.

Stepwise and multiple regression models revealed that WHtR and HC significantly predict agility performance in the OB group, explaining 29% of the shuttle-run test variability. In contrast, BMI was the sole significant predictor for the NORMW group. No significant predictors were identified for the overweight group. As expected, predictive potential differs between weight categories with central obesity markers (WHtR and HC) being the main predictors of agility. Intuitively, we would expect obese participants to have lower agility performance. Excess body weight, particularly abdominal obesity, can increase breathing work and reduce exercise capacity, particularly when lung function declines [33], prompting the inclusion of WHtR and HC in multivariate analysis. However, BMI is the best predictor of agility performance in the NORMW group. This study’s results are consistent with previous research [5] but differ from Prieto-González [2]. These discrepancies could be explained by the different characteristics of each population and the agility test performed.

The study found that WC and HC were significant predictors of push-up performance in the OB group, accounting for 38% of its variability. The OVERW group’s push-up variability was primarily due to weight and BMI, accounting for 24%, while the NORMW group’s weight was the only significant predictor, accounting for 10%. These findings suggest that WC plays a crucial role in predicting upper-body muscular endurance, particularly in individuals with higher body mass [34]. Supporting these results, Vaara et al. [35] demonstrated that push-up performance accurately measures body fat content, maximal aerobic capacity, and upper-body maximal strength.

The most salient result of the present study is the significant relationship between the BRI and the VO_2_max in all categories. Stepwise and multiple regression models revealed that BRI accounted for 56%, 46%, and 32% of VO_2_max variance in OB, OVERW, and NORMW groups, highlighting its predictive potential for cardiorespiratory endurance. From our stepwise regression modeling, we observed some interesting body category interactions between BRI and VO_2_max for OB (r = −0.75), OVERW (r = −0.59), and NORMW (r = −0.56) groups. This body roundness index, which accurately represents body fat distribution and visceral fat, has been suggested to be more closely associated with metabolic and cardiorespiratory disease complications, and all-cause mortality [16–18,36,37]. While scientific investigations linking BRI to physical fitness are still lacking, we can draw conclusions based on scientists’ and clinicians’ understanding of BRI and its relationship to health outcomes [15,17,18,36,37]. Thomas et al [38] found that higher visceral fat levels were linked to lower VO_2_max, regardless of BMI, suggesting the importance of fat distribution in determining cardiorespiratory endurance [38,39]. Excess body weight, particularly abdominal obesity, can increase breathing work and reduce exercise capacity, particularly when lung function declines [33]. Furthermore, as advanced by Krakauer and Krakauer [14], BRI is a better metabolic and cardiorespiratory risk than BMI, and may indirectly reflect an individual’s aerobic fitness potential. The current study indicates that BRI can effectively predict VO₂max performance among students, regardless of their physical composition.

The sit-and-reach test showed weak correlations with anthropometric measurements, with height and ABSI being the best predictors for normal weight. However, these parameters only explained 9% of performance variation, suggesting flexibility is not well predicted. Hung et al [40] found no significant correlations between adult sit-and-reach performance and BMI, WC, WHR, or WHtR. Prieto-González [2] found that flexibility was correlated only with abdominal girth, despite the number of anthropometric and health variables analyzed. Neuromuscular and biomechanical factors, including joint range of motion, muscle and tendon elasticity, limb length proportions, and nervous system tolerance to stretch, are likely to significantly impact flexibility performance. Excess abdominal fat may mechanically restrict forward trunk flexion, evidencing the observed correlation [2].

The regression models based on selected anthropometric factors had limited predictive capacity for physical performance tests like shuttle run, push-up, and sit-and-reach, suggesting that body measurements alone are insufficient predictors. However, VO₂max showed a stronger association with body composition, suggesting a more reliable link between anthropometric attributes and cardiovascular fitness.

The primary limitation of this study is the absence of an underweight group, which results in an incomplete representation of the full population spectrum. Additionally, the findings’ generalizability is constrained due to the sample being exclusively composed of young adult men. The cross-sectional design further limits the ability to draw causal conclusions; while correlations between variables can be observed, cause-and-effect relationships cannot be established. Furthermore, anthropometric factors such as body fat percentage and fat-free mass were not considered. Although the study identifies WC, HC, WHtR, BMI, and BRI as potential predictors of physical performance, it is important to recognize that fitness outcomes are influenced by a wide range of factors, including genetic, environmental, and behavioral variables, which were not included in this research. Future studies would benefit from a longitudinal approach, a broader array of fitness assessments, and a more diverse sample, including women and individuals from various age groups, to increase the applicability and depth of the findings.

## Conclusion

The study found that WRtR and HC significantly improved shuttle-run productivity in the OB group. In contrast, WC and HC, and WC and BMI significantly improved push-up productivity in the OB and OVERW groups. However, BRI was the most accurate indicator of VO_2_max performance, accounting for 56%, 46%, and 31% of the variance in OB, OVERW, and NORMW participants. The results should be considered in determining the appropriate anthropometric measures for assessing physical fitness in young adult males of different body categories. Additional researches are required to confirm these associations and examine the relationship between changes in BRI and changes in cardiorespiratory endurance.

## Supporting information

S1 File Human Participants Research Checklist. (DOCX)

S2 File Supporting Data. (XLSX)
